# Powdered tart cherry supplementation surrounding a single bout of intense resistance exercise demonstrates potential attenuation of recovery strength decrement with no definitive oxidative or inflammatory effect

**DOI:** 10.1186/1550-2783-12-S1-P25

**Published:** 2015-09-21

**Authors:** K Levers, R Dalton, E Galvan, C Goodenough, A O'Connor, S Simbo, N Barringer, S Mertens-Talcott, C Rasmussen, M Greenwood, R Kreider

**Affiliations:** 1 Exercise & Sport Nutrition Lab, Department of Health and Kinesiology, Texas A&M University, College Station, TX 77843, USA; 2 Department of Nutrition and Food Science, Texas A&M University, College Station, TX 77843, USA

## Background

Consumption of tart cherry juice has been reported to increase subsequent resistance exercise performance by reducing inflammation and oxidative stress that cause secondary muscle damage following initial bouts of resistance exercise. The purpose of this study was to determine if consumption of a powdered form of tart cherries derived from tart cherry skins (CherryPURE^®^ Freeze Dried Tart Cherry Powder) prior to and following intense resistance exercise increases subsequent performance while attenuating markers of inflammation and oxidative stress.

## Methods

23 resistance trained men (20.9 ± 2.6 yr, 14.2 ± 5.4% body fat, 63.9 ± 8.6kg FFM) were matched based on relative maximal back squat strength, age, body weight, and fat free mass. Subjects were randomly assigned to ingest in a double blind manner capsules containing a placebo (P, n = 12) or powdered tart cherries [CherryPURE^®^] (TC, n = 11). The lifters ingested the supplements one time daily (480 mg/d) for 10-d: 7-d pre-exercise, day of exercise, and 48-hr post-exercise. Subjects performed 10 sets of 10 repetitions at 70% of 1RM back squat exercises with 3-min recovery between sets, maintaining equivalent total work performed (p = 0.80) and average daily caloric consumption (p = 0.61) between groups. Isokinetic knee extension/flexion maximal voluntary contractions (MVCs) and fasting blood samples were taken pre-squat workout, 60-min following squat workout as well as after 24-h and 48-h of recovery and analyzed by MANOVA with repeated measures.

## Results

Powdered tart cherry supplementation seemed to attenuate the drop from pre-lift measures in 3-repetition summation of isokinetic flexion work (p = 0.21; d = 0.45, 0.28), extension work (p = 0.23; d = 0.45, 0.36), and all work (p = 0.15; d = 0.55, 0.37) through 60-min and 24-h of recovery compared to placebo as reported above by Cohen's d effect size, despite not being statistically significant. The overall MANOVA analysis revealed a significant Wilks' Lambda time (p < 0.001) interaction for all inflammatory markers, but no significant group × time pro-inflammatory (p = 0.30) and anti-inflammatory (p = 0.45) effects. Univariate measures for pro-inflammatory markers reported significant main time effects for TNF-α (p = 0.001), IL-1β (p = 0.30), IL-6 (p = 0.023), and IL-8 (p = 0.018). Univariate measures for anti-inflammatory markers reported significant main time effects for IL-4 (p = 0.001) and IL-7 (p = 0.033) with IL-13 trending toward significance (p = 0.055). No significant group × time effects were observed for any of the inflammatory markers, NT, or TBARS. Serum IL-1β levels were significantly lower in TC compared to P (p = 0.048). Delta changes were assessed at all three recovery time points from the pre-lift marker measures. The overall delta MANOVA analysis revealed a significant Wilks' Lambda pro-inflammatory interaction across time (p = 0.001) and an anti-inflammatory time interaction trending toward significance (p = 0.070), but no significant group × time pro-inflammatory (p = 0.44) or anti-inflammatory (p = 0.30) effects. TNF-α (p = 0.010), IFN-γ (p = 0.042), IL-1β (p = 0.031), IL-6 (p = 0.001), IL-8 (p = 0.025), IL-10 (p = 0.019), and NT (p = 0.018) demonstrated significant main effects on time, while IL-7 approached significance across time (p = 0.095). Serum IL-2 TC (p = 0.074) and IL-10 (p = 0.10) changes from pre-lift tended to be greater across the recovery time coupled with a tendency for IL-10 (p = 0.063) TC levels to also be greater compared to P. Contrarily, serum IFN-γ (p = 0.021) TC changes from pre-lift values were significantly smaller compared to P with specific differences at 24-h and 48-h post-lift.

## Conclusion

In accordance with previous TC juice supplementation research, the isokinetic performance results of this study indicate that short-term powdered TC consumption 7 days prior to, day of, and 2 days after a single bout of intense resistance exercise may help to attenuate the strength decrement over a 48-h recovery period. The seemingly better maintenance of strength during recovery with short-term powdered TC supplementation surrounding a single bout of resistance exercise did not, however, coincide with any definitive effect on markers of oxidative damage or inflammation. This may be due to the differences in resistance exercise metabolic demands, thus indicating the need for further mechanistic research.

